# Anatomy and Surgery

**Published:** 1866-01

**Authors:** 


### ABSTRACTS AND SELECTED ARTICLES.

### ANATOMY AND SURGERY.

*Strangulated Hernia treated successfully by Inflation of the Bowels, and shaking the patient while in an inverted position.*

Richard Griffin, Esq., in an article in the *British Medical Journal*, reports six cases of strangulated hernia treated successfully by the above method, after the failure of other remedies. The bowels were inflated by means of a bellows, the nozzle of which was introduced into the anus.

In one of the cases reported, the reduction was effected after strecoraceous vomiting had existed for three or four days. But the patient died from the diarrhœa induced by the large amount of drastic mogatives which had been administered. In four of the six cases, the hernia was reduced by inflation of the bowels alone; in the other two, this means failing, shaking the patient while in an inverted position was resorted to with success.

---

*Bromine as an Antidote to Zymotic Poisons—Its Application in the Treatment of Hospital Gangrene, Syphilis and Small-pox.*

The above is the title of an article by Dr. E. A. Clark, published in the *St. Louis Medical and Surgical Journal.*

Dr. Clark thinks that the use of bromine in the treatment of hospital gangrene, has developed the fact that it is an antidote to all animal poisons; and that iodine, though similar in its mode of action, is greatly inferior to bromine in the extent of its effects, especially in its local application. In defining the analogy of these medicines he compares them to the different preparations of bark, "which, in some instances," he says, "may be given in substance with excellent results and meet the indications in the case. Yet who would think of giving bark in substance; or cinchona in a case of pernicious intermittent, in place of quinine." * * * "Then the relative value of bromine and iodine, as therapeutic agents, can only be estimated by the comparative results." * * * * *

"Bromine also exerts an action on the blood in destroying zymotic poisons, which iodine does not possess to the same extent. The greater volatility of bromine also gives it an advantage in application which iodine does not possess, as in instances when the fumes of the medicine are required for disinfectant purposes."

Dr. Clark reports some cases of hospital gangrene successfully treated by him with bromine, and gives directions for its application so that it may prove effectual.

He says further: "In addition to the use of bromine in hospital gangrene, I have also used it as a local application in the treatment of syphilis; my experience with the remedy has not been sufficient, however, to warrant the expression of a positive opinion in its favor, as I have used it in only three cases, one of which was an indurated chancre, to which bromine was applied in substance the first time, and subsequently with the following solution:

| | | |
|---|---|---|
| ℞ | Bromine, | ʒss. |
| | Bromide of potassium, | ʒss. |
| | Aquae dist., | ℥ss. M. |

This solution was applied to the chancre once every other day, until four applications were made; during this time, however, the patient had taken mercury to the extent of procuring slight ptyalism, which was persevered in until the chancre had quite healed, requiring in all fourteen days." The other cases were soft chancre, to which bromine was applied at the same time that mercury was administered, followed by satisfactory results.

Dr. Clark used bromine, also, in two cases of small-pox with satisfactory results. It did not abort the disease, but modified it. (This agrees with our own observations of the action of this remedy in small-pox.—Eds. Jour.)

---

*United States Army Surgical Reports.*

The Surgeon-General has just published Circular Number Six for the information of the medical officers of the army. The circular comprises reports from Brevet Lieutenant-Colonel George A. Otis, U. S. Volunteers, having charge of the surgical, and Brevet Major J. J. Woodward, U. S. Army, having charge of the medical history of the rebellion. From the surgical report it appears that complete register of the wounded are in [illegible]se of preparation, in which over eighty-seven thousand [illegible] of wounds, and seventeen thousand surgical operations have been recorded up to September, 1865, the work of registration being still very far from complete. The material collected is enormous, and embraces a mass of facts which on many subjects exceed in number and value all previous observations in this field.

In the late war the monthly reports from a little more than half the regiments in the field, give for the year ending June 30, 1862, an aggregate of 17,496 gun-shot wounds. The report from rather more than three-fourths of the regiments for the year ending June 30, 1863, give a total of 55,974 gun-shot wounds. The battle-field lists of wounded for the years of 1864–65, include over 114,000 names.

The surgical specimens of the Army Medical Museum number 5480, and not only in specimens of recent injuries, but in illustration of reparative progress after injury, of morbid processes, of the results of operations and surgical apparatus and appliances, this institution is richer, numerically at least, than the medico-military museums of France or Great Britain.

The medical staff that served in the late war was composed of a Surgeon-General, one Assistant Surgeon-General and Medical Inspector-General, 16 medical inspectors, 170 surgeons and assistant surgeons of the regular army, 362 volunteer staff surgeons and assistant surgeons, 3000 regimental surgeons and assistant surgeons of volunteers, 2500 acting assistant surgeons, or physicians serving under contract, and six medical storekeepers.

The second report, by Major Woodward, contains an outline of the material collected for the medical branch of the history. It embraces all the information possible with regard to the sickness and mortality of the army during the war, and especially whatever related to the nature and causes of those afflictions which were the chief occasion of death and disability. The mortality from disease alone was forty-eight and seven-tenths per one thousand of mean strength for the first year of the war, and sixty-five and two-tenths for the second. The total number of deaths from disease reported for the first year was 14,183, and 42,010 for the second. These figures do not include those who died while absent as prisoners of war or after having been discharged the service for disability. The number constantly sick was about ten per cent. of the strength. The total number of cases treated by the medical department, including wounds and injuries, was 878,918 during the first year, and 171,183 during the second. The most fatal disease was camp fever, of which there were 213,260 cases, and 19,459 deaths, during the two years. Next come diarrhœa and dysentery, 725,675 cases and 11,560 deaths. The inflammation of the respiratory organs, 304,284 cases and 8090 deaths. Venereal diseases were much less frequent than the experience of other armies would have led us to expect, still eighty-four men in every thousand suffered during the first year, and sixty-five during the second—the total number of cases being over thirty-nine thousand. Twenty-eight thousand six hundred and twenty discharges for disability were reported during the first year, or about nine per cent. of the strength of the army.

It appears that at the medium there were 202 general hospitals, 136,894 beds for patients. During the war over a million were treated in these, of whom but one in twelve died. Dr. Woodward says, never before in the history of the world has the mortality in military hospitals been so small, and never have such establishments so completely escaped from diseases generated within their walls. Complete reports for the first year of the war from troops in the field and in garrison, represent an average strength constantly present during the year of 281,117 men; in hospital constantly present, 9759 men; total, 290,936, among whom were 14,183 deaths from disease. The number of deaths recorded is much less than the real nnmber, as it does not include prisoners of war and other absentees. For the second year in field and garrison, 598,821; in hospital, 45,687; total, 644,508; of whom there were 42,010 deaths from disease. These mortality rates from disease are much smaller than is usual with armies in a time of war, and are much less than those of the allied armies in the Crimea, of our own army in the Mexican war. The proportion of deaths from disease for the third and fourth years was rather diminished.—*Despatch to Daily Advertiser.*

---

### *An Operation for Fistula in Ano Two Hundred Years Ago.*

M. Le Roi, in his *Curiosities Historiques*, gives the following interesting account of a "grand operation" performed on Louis XIV.:

On the 18th of November, 1686, Versailles was astounded with the news that Louis XIV. had undergone the "*grand operation*," as it was then called, for fistula *in ano*. An abscess at the margin of the anus of the king had been discovered in February, 1686; a Felix de Tassy, his chief surgeon, at once proposed to open it. But, as Dionis remarks, "the deference to opinion necessary for a cure is not always found among the great." A thousand *infallible* remedies were immediately proposed; and of these, a plaster made by Madame de la Daubiere was selected as preferable to the lancet of the surgeon; and madame herself, as the chronicle tells, assisted in its application. The plaster, however, was removed five days later, having only increased the king's sufferings; and it was at last resolved, on the 23d, that the abscess should be opened. But, contrary to the wishes of Felix, caustic was used for the purpose, instead of the knife. The result was that, when the caustic slough fell off, "the pus ran out of a small hole." Soon afterwards it was discovered that the fistula communicated with the interior of the gut; and an operation for its cure was therefore necessary.

Hereupon the king was again overwhelmed with the promises of infallible curers of *tumors*. Louvois, the minister, however, being in some sense responsible for the life of the king, would not allow any of their remedies to be applied to the king without having them previously tried on others. Thus, for example, the use of the waters of the Bareges having been recommended, and having taken the fancy of the king himself, four persons with fistula were sent to Bareges, under the charge of Gervais, surgeon of La Charite, who had a great reputation in the cure of tumors. They were treated in a variety of ways, internally and externally, with the waters, for a long time; but the result was, says Dionis, that at the end they were no nearer a cure than when they started at Bareges. Next came a court lady, who reported that she had been cured of a fistula at the waters of Bourbon. Consequently four other patients with fistula were sent there, under one of the king's surgeons; but they also returned uncured. Still Louvois was besieged with promises of a cure; and, not wishing to throw away a chance, had several rooms in his ministerial hotel at Versailles fitted up for the reception of all fistula *in ano* patients who were willing to have remedies tried upon them. The experiments were carried on for a length of time; but all the *infallible* waters, ointments, etc., turned out complete failures.

Of all these proceedings the king was informed by Louvois and Felix, who urged upon him that all attempts to cure the fistula without operation were in vain. Before finally deciding, the king sent for Bessieres, who was then in great repute at Paris; and by him was told that "all the remedies in the world would do nothing without an operation." Thereupon the king determined to undergo the operation. But *what* operation? At that time there lived at Paris one Lemoyne, who had great repute as a curer of fistula. Of him Dionis writes:

"His method consists in the use of a caustic, spread over a little plug, and introduced into the opening; this plug is enlarged day by day, so as to destroy all callosities and sinuses. By this process, and with plenty of patience, he cured many a fistulæ. He died old and rich; and he made the people pay well; and in this he was right, as the public only esteem those things which cost them dear. All patients who dreaded cutting fell into his hands; and, as the number of cowards (*poltrons*) is always great, he never wanted for practice."

The ligature was the plan most in use; but Felix preferred the knife. Felix, therefore, was called upon to describe to the king the entire history of these different remedies. Caustic, he told him, produced continual pain for five or six weeks. The ligature required a long time to cut its way through, and also needed frequent tightening, and so constantly produced pain. The pain of incision was, he admitted, sharper, but then it was only for a moment; and the cure for incision was certain and rapid, and this could not be said of either the caustic or the ligature. Felix's arguments, supported by those of d'Aquin, Fagon, and Bessieres, decided the king for incision. Felix was a bold man; for at that time the operation by the knife was looked upon as a great and terrible affair. But Felix was not an ordinary man. He was the son of the king's surgeon, Felix de Tassy; had been carefully educated by his father, in hopes that he would become his successor; and at an early age was celebrated as a skillful surgeon. In 1676 he, in fact, became first surgeon of the king. But, while experiments of the "curers" were being tried, he read everything which had been written on the subject; and, what is more, operated on all the patients having fistula *in ano* who were received into the Paris hospitals and into La Charite at Versailles. When, therefore, the king at length resolved upon the operation, Felix had become a master in its performance.

Felix used a modification of Galen's syringotome. It was a very narrow curved knife, terminated by a stylet several inches long. The cutting part of the blade was covered with a silver sheath. This instrument was introduced through the fistula into the rectum and then brought out the anus. The sheath was then gently withdrawn; and the knife, now laid bare, held by the hand and by its stylet end, was at once made to cut its way out. This knife received the name of the *Royal Bistoury.*

The operation was performed on November 18th, 1686. The king had kept his intention a secret. He came to Versailles on the 15th; and on the 17th he rode out publicaly on horseback. On the 18th, at five o'clock in the morning, the apothecaries administered a lavement. A little before seven, Louvois brought Madame de Maintenon to the king, who was found engaged with Pere de la Chaise, his confessor. In the Cabinet des Bassans were assembled Felix, d'Aquin, the king's chief physician, Fagon, Bessieres, the four royal apothecaries, and Laraye, Felix's pupil. At seven o'clock they entered the king's room. Louis XIV. made Felix show and explain to him the instrument, etc.; and then with perfect confidence, and most composedly, (as we are assured,) placed himself in Felix's hands. The operation was performed in the manner above indicated; and then, with eight cuts of scissors, Felix removed the callosities which were exposed when the inscision was made. Louis bore the operation without a cry or a word. A large plug of charpie, covered with oil and yolk of egg, was then forced into the wound.

Consternation seized all the courtiers when they heard that the king had undergone this *dangerous* operation. For the first few days afterwards, things went on well; but, on December 7th, it was found necessary to destroy the new cicatrix, and to lay bare the fistula to its base. After this second proceeding, the operation succeeded.

Felix was well rewarded. His fee was 50,000 crowns, and the estate of Mouhineaux, estimated at a like value. D'Aquin received 100,000 *livres;* Fagon, 80,000 *livres;* the four apothecaries, each 12,000 *livres;* and the pupil Laraye (who was not forgotten), 400 *pistoles*—the sum paid amounting altogether to about £40,000! Felix's practice now naturally became very large; for, as Dionis says:

"Fistula *in ano* has become a fashionable disease since the operation was practiced on the king. Those who had before, through shame, concealed the disease, now made it public; and many went to Versailles to undergo the operation, because they knew that the king made inquiries about all operations of the kind. Some even, who had simply hæmorrhoids, or a slight discharge, were angry when they were told there was no necessity for an operation."

"Such," says M. Le Roi, "is the history of the operation performed on Louis XIV. Thanks to the happy initiative of Felix, the method of operating by incision was again brought into honour, and has since been generally adopted. A man, indeed, may now-a-days successfully practise the operation without being first surgeon of the king—so simple has the operation become."—*Brit. Med. Journ.*, Sept. 23, 1865.

